# Machine Learning Methods for Classifying Human Physical Activity from On-Body Accelerometers

**DOI:** 10.3390/s100201154

**Published:** 2010-02-01

**Authors:** Andrea Mannini, Angelo Maria Sabatini

**Affiliations:** ARTS Lab, Scuola Superiore Sant'Anna, Piazza Martiri della Libertà, 33–56124 Pisa, Italy; E-Mail: a.mannini@sssup.it

**Keywords:** wearable sensors, accelerometers, motion analysis, human physical activity, machine learning, statistical pattern recognition, Hidden Markov Models

## Abstract

The use of on-body wearable sensors is widespread in several academic and industrial domains. Of great interest are their applications in ambulatory monitoring and pervasive computing systems; here, some quantitative analysis of human motion and its automatic classification are the main computational tasks to be pursued. In this paper, we discuss how human physical activity can be classified using on-body accelerometers, with a major emphasis devoted to the computational algorithms employed for this purpose. In particular, we motivate our current interest for classifiers based on Hidden Markov Models (HMMs). An example is illustrated and discussed by analysing a dataset of accelerometer time series.

## 1. Introduction

The availability of a system capable of automatically classifying the physical activity performed by a human subject is extremely attractive for many applications in the field of healthcare monitoring and in developing advanced human-machine interfaces. By the term physical activity, we mean either static postures, such as standing, sitting, lying, or dynamic motions, such as walking, running, stair climbing, cycling, and so forth. More precisely, we distinguish in this paper between primitives, namely elementary activities like the ones just mentioned, and composite activities, namely sequences of primitives, e.g., sitting-standing-walking-standing-sitting, in as much the same way as we distinguish between words and sentences in a spoken language.

The information on the human physical activity is valuable in the long-term assessment of biomechanical parameters and physiological variables. Think, for instance, of the limitations when the metabolic energy expenditure of a human subject is estimated using indirect methods: serious estimation errors may occur when wearable sensor systems composed of motion sensors, such as accelerometers, are used without any regard to what she/he is actually doing [1,2]. The information on the physical activity is also valuable as a source of contextual knowledge [3]. Provided that this information is available, the human-machine interaction would be more complex and richer [4]. In robotics, several applications which demand some capability by the robot of recognising the user’s intent are, for instance, in the field of rehabilitation engineering, where smart walking support systems are currently developed to assist motor-impaired persons and elderly while they attempt to stand or to walk [5–7]. Mostly, the physical interaction between the user and the walking aid takes place through handles instrumented with force/torque sensors [8]; the signals acquired from these sensors can be exploited not only for guidance purposes, but also for gaining some form of contextual awareness [9]. In some cases, proximity/range sensing or even inertial sensing are used to detect incipient gait instabilities of the user [10,11], in order that a prompt response by the walking aid controller may be issued in the attempt, e.g., to minimise the risk of fall [11].

**Figure 1.**
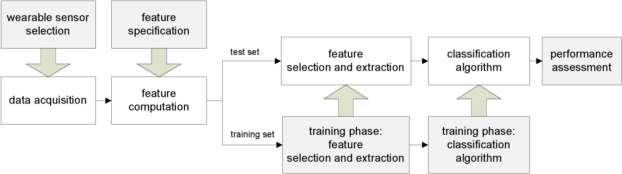
Conceptual scheme of a generic classification system with supervised learning.

In this paper the most common approaches to automatic classification of human physical activity are introduced and discussed. In regard to the problem stated above, the main steps regarding sensor selection, data acquisition, feature selection, extraction and classification are reviewed by tracing the diagram of Figure 1. As for the machine learning techniques needed for classification, particular emphasis is given here to Markov modelling. Albeit identification of context without requiring external supervision seems better suited to make intelligent systems [12], most current approaches in the field are based on using supervised machine learning techniques. The use of Hidden Markov Models (HMMs) is attractive, although they are known potentially plagued by severe difficulties of parameter estimation. In this paper we exploit an annotated dataset of signals from on-body accelerometers in order to test several classification algorithms, including HMMs with supervised learning. Results of a validation study are presented.

## 2. Methods for Automatic Classification of Human Physical Activity

### 2.1. Wearable sensors and data acquisition

The first important aspect to be considered in building a system for automatic classification of human physical activity concerns the choice of sensors. Wearable sensors should be small and lightweight, in order to be fastened to the human body without compromising the user’s comfort and allowing her/him to perform under unrestrained conditions as much as possible. Although ultrasonic or electromagnetic localisation systems [13], opto-electronic marker-based [14] or markerless systems [15] all represent possible choices, common to all of them is the limitation that external sources are generally required, which restricts their sensing range, and lead to additional difficulties, *i.e.*, occlusions and interference. Inertial sensors are an interesting choice, since they are self-contained, immune to occlusions and interference, although the processing is seriously limited by sensor noise and drift, which prevent them from delivering accurate position/orientation data beyond few seconds or minutes, unless a very sophisticated and complex filtering is applied to raw sensor signals [16]. This is true especially for those technologies that are the most promising in terms of cost, burden, and power consumption, namely microelectromechanical systems (MEMS) accelerometers and gyros [17]. Most features of MEMS inertial sensors seem to fit well with the requirements of motions sensors for biomechanical applications, which motivates their growing use and great interest amongst the practitioners in the field [18]. The main reason for their widespread acceptance is that they allow, in principle, to perform quantitative functional assessment in unrestrained conditions: tested subjects do not easily incur in those behavioural artefacts which are typical when standard motion analysis technology is used in a specialised laboratory [19].

Historically, accelerometers entered the biomechanical arena well in advance to gyros. Few pioneering contributions [20,21] highlight the idea that the acceleration field of any *rigid* part of the human body can be measured and reconstructed by user-worn accelerometers, which may ultimately lead to compute the pose and orientation of this part. Interesting works reported in the literature over the years concern, among other aspects, the estimation of head motions [22], and the estimation of spatio-temporal parameters of gait [23]. More recently, the availability of miniature MEMS vibrating gyros has fostered several research reports, where they are used for applications in gait analysis, either alone or in combination with accelerometers [24,25]. Moreover, recent developments concern the integration of triads of accelerometers and gyros with mutually orthogonal sensitive axes within three-dimensional strap-down inertial navigation systems that are proposed for applications in virtual reality, pedestrian navigation, robotics, and so forth [18]; oftentimes, they are used in combination with additional navigation aids, including Global Positioning System (GPS) receivers and magnetometers, to provide position/velocity and attitude navigation data [26].

Interestingly, using accelerometers is also commonplace in many other biomedical applications, such as tremor analysis [27], assessment of physical activity [28] and quantification of metabolic energy expenditure [29], where the computational techniques of interest do not require error-prone procedures for nonlinear differential equations systems integration from noisy data and uncertain initial conditions. In these applications, the computational techniques of interest have to do mainly with the implementation of machine learning algorithms, which are often aimed at performing nonlinear multivariate regressions and pattern recognition.

### 2.2. Feature evaluation

A pattern recognition machine does not perform its classification tasks working directly on the raw sensor data. Usually, the classification is pursued after that a data representation is built in terms of feature variables. The choice of features with high information content for classification purpose is both a fundamental step in the development of any pattern recognition machine and a highly problem-dependent task.

An accelerometer—the sensor of main interest in this paper—measures the projection along its sensitive axis of the specific force *f* applied to the body it is fastened. The specific force additively combines the linear acceleration component *a*, due to body motion, and the gravitational acceleration component, *g*—both projected along the sensitive axis of the accelerometer [18]. In common parlance, the high-frequency component, aka the AC component, is related to the dynamic motion the subject is performing, e.g., walking, hand weaving, head shaking, and so forth, while the low-frequency component of the acceleration signal, aka the zero-frequency (DC) component, is related to the influence of gravity, hence it can be exploited to identify static postures [30]. This is a key point in specifying the feature variables of interest, which are usually evaluated from the raw sensor data within sliding windows with finite and constant width, henceforth called data frames.

Although the choice of features is problem-specific, and different researchers may pursue different approaches for their identification and computation [31], the features proposed in this paper are quite popular amongst the practitioners in the field [32].

The DC component of acceleration is estimated by taking the signal average from the data samples within each frame. Since each accelerometer axis provides a data frame, the DC component feature vector can be conveniently used to get an idea about how the body is oriented in space with respect to the gravity direction. The DC component is thus well suited to classify postures.

Simple statistical descriptors, such as the variance, are widely used; the variance is computed by taking the average of the squared detrended data samples within each frame. The signal energy and the distribution of signal energy over the frequency domain are other popular choices. Frequency-domain features can be derived from the coefficients of time-frequency transforms, like the Short Time Frequency Transform (STFT), the Continuous or the Discrete Wavelet Transform (CWT, DWT) [32–34]. Beside their role as motion signatures, energy features can also be used to assess the strength of the motor act, the importance of which in assessing the energy expenditure incurred by the subject is well recognised in the literature [14,35].

The frequency-domain entropy is helpful in discriminating primitives that differ in complexity. As a matter of fact, walking and cycling can be difficult to discriminate based on the DC component and energy features; however, the walking entropy turns out to be much higher than the cycling entropy, mainly because of the foot impacts with ground occurring during walking, which give rise to the distinctive high-frequency coloured noise-like signatures typically observed in the signals from on-body accelerometers. In this paper, the coefficients of the STFT transform are used to compute the frequency-domain entropy [32].

The correlation coefficients between each pair of accelerometer signals are also useful features. They are obtained by computing the dot product of pairs of frame vectors, normalised to their length, and are highly helpful in discriminating activities that involve motions of several body parts [36].

### 2.3. Feature selection and extraction

When the dimension of the feature space is high, learning the parameters of a classifier becomes a difficult task, especially when the size of the training set is small (the curse of dimensionality). Usually, one individuates, empirically or based on theoretically sound considerations, as many features as needed to deal with the classification problem at hand. The available dataset is then divided into a training set and a test set. As a rule of thumb, the *n/d* ratio between the number of instances *n* available in the training set and the dimension *d* of the feature-space must be at least ten. Since the achievable performance of a classification algorithm tends to critically depend on the dimension of the feature space, methods for reduction of dimensionality are oftentimes considered in developing the classifier. These methods are based on two main approaches: feature selection and feature extraction [37].

The feature selection approach consists of detecting and discarding the features that are demonstrated to minimally help to cause a correct response by the classifier. The identification of the optimal feature set is not always feasible because of the high computational costs connected to searching through an inordinate number of *m*-dimensional subsets (1≤ *m ≤ d*). Usually, the feature selection step is implemented via sub-optimal search algorithms, such as, for instance, the branch-and-bound search, the sequential forward-backward selection (SFS-SBS), the Pudil algorithm based on a sequential forward-backward floating search (SFFS-SFBS). Of particular interest are the sequential search algorithms; these are iterative procedures that add and/or remove a fixed or variable number of features at each step, while assessing the effects of these modifications according to pre-defined quantitative criteria. One of such criteria is based on computing the Euclidean distances between each pair of feature vectors in the training set (k-Nearest Neighbour, k-NN). The ratio between inter-class and intra-class distances is then maximised across the various feature subsets. Other criteria can be devised by analysing the classifier output: the computational costs of these criteria are generally high, however the assessment procedure turns out to be oriented at the very goal of the classification process.

The feature extraction approach revolves around the idea that data representations can be constructed in subspaces with reduced dimension, while at the same retaining, if not increasing, the discriminative capability of the new set of feature variables [37]. This may happen at the expense of losing their physical meaning. By far, the most popular feature extractor is the principal component analysis (PCA) or Karhunen-Loève transform, that transforms feature variables into a smaller number of uncorrelated variables called principal components. In this approach, upon eigenvalue analysis of the *d × d* data covariance matrix, the new feature vectors are the eigenvectors associated to the *m* largest eigenvalues. Another approach, similar in concept, is the independent component analysis (ICA), often applied in problems of blind source separation: a PCA is followed by a data whitening transformation, with the aim of finding the independent components of a process, namely the attempt is made to reduce the process to its additive components [34].

Feature selection and feature extraction are not necessarily cascaded in some predefined order. Oftentimes, for instance, a feature selection algorithm is either applied to data that have been previously subjected to dimensionality reduction by feature extraction, or without a successive extraction step.

### 2.4. Taxonomy of classifiers

A taxonomy of classifiers can be built according to different criteria [37]. First, a distinction is between supervised and unsupervised classifiers. In supervised classifiers, the training set is labelled, namely the membership of the feature vectors to a given class is known to the system in advance. According to an unsupervised approach, only the number of classes *C* is known, and the system responds to the instances in the training set by assigning a label to each of them. Second, single-frame and sequential approaches to classification can be distinguished. A single-frame classifier works by assigning a label to each data frame it receives at its input, in isolation from the history of previous assignments. Conversely, a sequential classifier takes the *past* classifications into account in order to orient the decision on the current feature vector. The classifiers can be further divided according to three main approaches: probabilistic, geometric, and template matching. Table 1 summarises the state of the art for classification of human physical activity; succinct information is also included, as for sensor and feature type and number; method of classification; number of activities and tested subjects; accuracy of classification.

In accordance to the rules of the probabilistic approach, a feature vector **x** is classified as belonging to the class which turns into the maximum value of the class-conditional PDFs *p*(**x***|C_i_*), *i* = 1, …, *C*. The class-conditional PDF denotes how likely is a feature vector to belong to a given class. An example of probabilistic classifiers is the optimal Bayesian classifier. Since class-conditional PDFs are usually not known, suboptimal implementations have to be considered, e.g., naive Bayesian, logistic, Parzen and Gaussian Mixture Model (GMM) classifiers [32]. The Parzen classifier provides an estimate of the class-conditional PDF by, e.g., applying a kernel density estimator to the labelled feature vectors in the training set, while a GMM classifier estimates class-conditional PDFs using mixtures of multivariate normal PDFs [38].

In the geometric approach the classification is performed based upon the construction of decision boundaries in the feature space that specify regions for each class. Decision boundaries are constructed during the training session via iterative procedures or geometrical considerations. As a matter of fact, Artificial Neural Networks (ANN) are based on iteratively tessellating the feature space [33], whereas k-Nearest Neighbour (*k-*NN) classifiers, and Nearest Mean (NM) classifiers work directly on the geometrical distances between feature vectors from different classes [39]. Finally, Support Vector Machines (SVM) classifiers are geometric-based classifiers that construct boundaries maximising the margins between the nearest features relative to two distinct classes [14]. Another popular approach is the threshold-based classifier, as noted in Table 1.

A carefully handcrafted setting of thresholds is required in order to separate the various classes under examination. For instance, a threshold based on an energy-related feature, or simply the data variance, helps discriminate between presence and absence of motion. The main disadvantage of this approach is its potential sensitivity to intra and inter individual variations and to the precise placement of sensors. In this sense extensive handcrafting of classifier parameters is believed to be detrimental for achieving good generalisation properties of the classifier itself [46].

The template matching approach is based on the concept of similarity between observed data and activity templates, either defined by the designer or obtained during the training session. The editing and condensing techniques, customarily applied to *k-*NN classifiers, can be useful for defining the templates. A classification that is based on individual reference patterns appears to be less susceptible than threshold-based classification, although careful sensor placement is critical for achieving good test-retest reliability. Applications of the template matching approach can be found, e.g., in [19]. In spite that they are widely used in classifying human physical activities, threshold-based and template matching methods are not tested in this paper.

Finally, there exist so-called binary classifiers, where the classification process is articulated in several different steps. At each step, different strategies, based on either threshold-based or template-matching detectors, are followed to reach a binary decision. For instance, in hierarchical binary decision trees each node is capable of discriminating between two states, and the classification becomes progressively more refined as the tree is descended along its branches [28].

### 2.5. Background on Markov models and Hidden Markov Models

**Figure 2.**
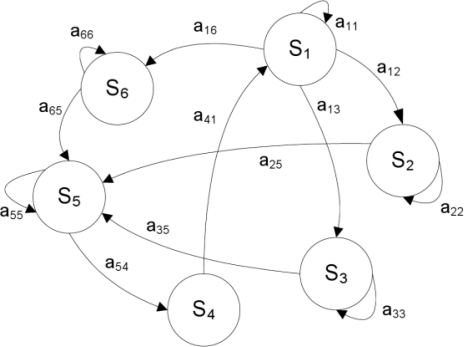
Graphical representation of a six-state Markov chain: the nodes are the states of the chain; the oriented arcs between nodes denote state-to-state transitions, including self-transitions.

Although the single-frame methods are quite widespread for classification of human physical activity, a possibly better way to deal with this problem is to exploit the decisions taken by the classifier in the past (sequential approach to classification). If we turn our attention to a sequential classification approach, a composite activity (motor sentence) can be conveniently viewed as the result of chaining a number of primitives (motor words). The knowledge about the way humans organise the functional tasks they are involved in during their daily life (motor language) can help describing the statistical properties of this chaining process. The sequential approach calls quite naturally for Markov modelling [48]. Henceforth, we assume that a composite activity can be modelled as a first-order Markov chain, composed of a finite number *Q* of states *S_i_*; each state accounts for a primitive. The time evolution of a first-order Markov chain is governed by the following quantities:

- prior probability vector **π**, with size (1 × *Q*); it is composed of the probabilities *π_i_* of each state *S_i_* of being the state *X* at the initial time *t*_0_:
  (1) $$\pi_{i}=\text{Pr}[X(t_{0})=S_{i}],\;\;\;i=1,...,Q$$
- transition probability matrix (TPM) **A**, with size (*Q* × *Q*), whose elements *a_ij_* are the probabilities of transitions from the state *S_i_* at time *t_n_* to the state *S_j_* occupied at time *t_n+_*_1_, as schematically depicted in Figure 2 for a six-state Markov chain:
  (2) $$a_{ij}=\text{Pr}[X(t_{n+1})=S_{j}|X(t_{n})=S_{i}],\;\;i,j=1,...,Q$$

Elementary considerations of probability calculus yield the following constraints for the transition probabilities:

(3) $$a_{\mathrm{ij}}\geq 0,\;\;\sum_{j=1}^{Q}a_{\mathrm{ij}}=1$$

The prior and transition probabilities needed to create the Observable Markov Model (OMM) (π, A) associated to the Markov chain can be empirically determined based on observations of the activity behaviour of a subject. If the TPM and the state at the current time are known, then the most likely state that will follow is probabilistically determined. In a more practical sense, each primitive can only be observed through a set of raw sensor signals (the measured time series from on-body accelerometers, in the present case). We would like to infer the hidden state from the available noisy observations, and to trace the time history of how the primitives have evolved up to the present time, in order to estimate the composite activity. In other words, the states are hidden and only a second-level process is actually observable. The observable outputs are called emissions.

If the assumption is made that the emissions are discrete, an alphabet **Ω** containing a finite number *W* of possible emissions *Z_i_, i* = 1, …, *W* is dealt with. The statistical model is called Hidden Markov Model (HMM); its specification requires a *Q* × *W* stochastic matrix that contains the probabilities *b_ij_* of getting an emission *Z_j_* at time *t_n_* from the state *S_i_*:

(4) $$b_{\mathrm{ij}}=\text{Pr}[Z(t_{n})=Z_{j}|X(t_{n})=S_{i}]$$

where:

(5) $$b_{ij}\geq 0\;\sum_{j=1}^{W}b_{ij}=1$$

Finally, an HMM is modelled by a parameter set ***λ*** that accounts for prior, transition and emission probabilities:

(6) λ=(π,A,B)

If the emissions are continuous, continuous PDFs are to be assigned, instead of probability mass functions (*continuous emissions* HMM, aka cHMM). The most common approach to the problem of modelling continuous emissions is parametric. A given distribution family is assumed for the emissions, and the parameters associated to the family are used to fully specify them. For instance, for a Gaussian cHMM we have:

(7) $$b_{j}=\sum_{m=1}^{M}c_{jm}N(\mu_{jm},\Sigma_{jm}),\;\;\;\;\;\;j=1,...,Q$$

where:

(8) $$\sum_{m=1}^{M}c_{jm}=1,\;\;\;\;j=1,...,Q$$

A mixture of *M* multivariate normal distributions *N*(**μ***_jm_*, Σ*_jm_*) with mean value **μ***_jm_*, covariance matrix **Σ***_jm_* and mixing parameters *c_jm_* is used to model the emissions from each state in the chain.

The HMM modelling framework requires that three main problems are solved, (a) thru (c): given an observation sequence **Z** = [*Z*(*t*_1_)*Z*(*t*_2_)…*Z*(*t_T_*)] and a model **λ**, evaluate: (a) the conditional probability *P*(**Z**|**λ**); (b) the most likely sequence of states **X** = [*X*(*t*_1_)*X*(*t*_2_)…*X*(*t_T_*)] occupied by the system; (c) identify the parameters of the model **λ**. The Viterbi algorithm is the most widespread solver of problem (b) and the Baum-Welch algorithm is popular for tackling problem (c). An excellent reference source for HMMs and algorithms for their learning and testing is [49].

### 2.6. HMM-based sequential classifiers

Currently, HMMs are applied in a large number of pattern recognition problems. For many years, speech recognition has been considered the killing application for HMM [49]. More recently, other applications have been investigated with remarkable achievements, just to mention a few of them, in developing systems for hand gesture recognition [50], sign language recognition [51], and functional assessment of human skills [52]. Specific applications to classification of human physical activity as pursued in this paper are relatively scarce [53]. Indeed, they seem to be more elusive as compared with the previous ones, in the face of the great variety of human motor behaviours [32]. Nonetheless, it is tempting to assume that primitives combine in time to form a composite activity as prescribed by a simple Markov model.

In this paper we propose to build a sequential classifier composed of a Gaussian cHMM. A potential problem with this approach is the huge number of parameters we need to estimate. In fact, a Gaussian cHMM trained in a *d*-dimensional feature space, *Q* primitives to be classified and *M* components for each mixture requires the specification of the following parameters:

- **π**, prior probability vector, 1 × *Q*;
- **A**, transition probability matrix *Q* × *Q*;
- **μ**, set of mean value matrices, *Q* × *M* × *d*;
- **Σ**, set of covariance matrices, *Q* × *M* × *d* × *d*;
- **C**, set of mixing parameters, *Q* × *M*.

Suppose that the training set presents only a relatively limited number of examples. A sensible approach to deal with the difficulty of parameter estimation may be to train, separately, different subsets of them. We propose to train the transition parameters, *i.e.*, **π** and **A** separately from the emission parameters, *i.e.*, **μ**, **Σ**, and **C**, by exploiting the annotations available in the dataset. As for the transition parameters, since their labelling is known, the composite activities in the training set are assumed generated by a *Q*-state OMM, the state and transition probabilities of which can be estimated by event counting. The emission parameters specify the Gaussian multivariate PDFs in the same way as the class-conditional PDFs are specified in probabilistic classifiers, such as, for instance, GMMs. As a whole, we refer to this initialisation phase as the first-level training phase. The values of the parameters estimated during the first-level training phase can be further refined by on-the-fly runs of the Baum-Welch algorithm (second-level training phase); this trick may help adapting the cHMM behaviour, in particular, to unexpected TPM changes.

**Figure 3.**
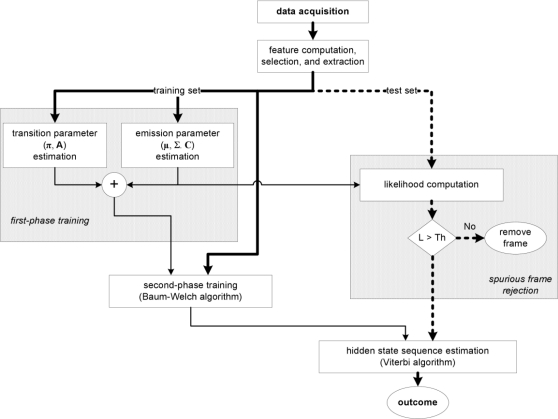
Block diagram of the developed cHMM-based sequential classifier.

Finally, an interesting feature of the classifier we have developed resides in its capability of managing spurious data. One difficulty for the classifier is in fact when activity primitives are presented during operation, and examples of them are not included in the training set. Our approach to deal with this problem consists of computing the likelihood of each feature vector, given the GMM structure that models the cHMM emissions. A simple threshold-based detector enables to flag anomalous feature vectors, preventing them from being actually presented to the classifier. Figure 3 shows the block diagram of the sequential cHMM-based classifier; in the figure we also indicated the block for spurious frame rejection.

## 3. Validation Study

At the time being, we are developing a wearable sensor system for indoor-outdoor pedestrian navigation, which embodies the following sub-systems: an on-body network of four tri-axial accelerometers, an on-foot fully integrated Inertial Measurement Unit (IMU) that includes a triad of magnetometers, and finally a waist-worn GPS receiver. Since the hardware and firmware components of this system are currently undergoing their production phase, the validation of the classification methods studied in this paper is based on analysing a dataset of acceleration waveforms, made available to us by Prof. Intille and associates at MIT [32].

### 3.1. Dataset for physical activity classification

**Figure 4.**
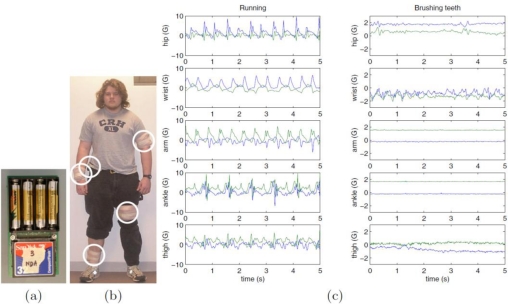
Experimental setup for the acquisition of the selected dataset (courtesy of Ling Bao and Stephen S. Intille © 2004 IEEE).

The classification methods were applied to the dataset described in [32]. Acceleration data, sampled at 76.25 Hz, were acquired from five bi-axial accelerometers, located at the hip, wrist, arm, ankle, and thigh. The original protocol was based on testing 20 subjects, who were requested to perform 20 activities (Figure 4). In this paper, 13 subjects were randomly selected for further analysis, in order to ease the development work. Moreover, because of our interest for personal navigation based on on-foot inertial sensing, we considered just the seven activities shown in Table 2, which involved primarily the use of the subjects’ lower limbs.

Since the research goal in [32] was exclusively to test single-frame classifiers, the available data for each subject concerned acceleration time series that were known to correspond to each primitive. Their work was thus at the level of motor words. Our validation study of single-frame classifiers followed their approach, although we opted for a subject-specific training, *i.e.*, a distinct classifier was trained for each individual subject. As for the cHMM-based sequential classifier, we built a *Q*-state OMM with known model (**π**, **A**) in order to generate motor sentences from the vocabulary of motor words in Table 2 (*Q* = 7). The simulation of a composite activity by a single subject (virtual experiment) was made by associating, for each tested subject, one data frame to each OMM state. The associated data frame was randomly sampled (with replacement) from the maximum number *N* of frames available in the reduced dataset for each primitive and subject (18 ≤ *N* ≤ 58). A number *S* = 20 of virtual experiments was synthesised, each of which composed of *T* = 300 data frames. A subset of *P* virtual experiments was included in the training set.

The procedure of synthesising virtual experiments in the manner described above implied the existence of clear-cut borders between data frames associated to different primitives, which were managed by data cropping in creating the original dataset [32]. Of course, real-life composite activities would be more complex, due to, say, *fuzzy* postural transitions in the data. In the attempt to get a more realistic picture of the cHMM-based sequential classifier performance, data frames from the original dataset not included in the reduced dataset were thus randomly interspersed in the tested data sequences generated by the OMM, in variable proportions, from null to 1:3 (max.). The resulting *garbage* was managed in our system by labelling data frames as spurious if their likelihood given the GMM structure that models the cHMM was below a properly settled threshold, as described in Section 2.6.

### 3.2. Feature vectors

The feature vectors were built from 50%-overlapping sliding windows with 512 samples. Since the sampling frequency was 76.25 Hz, each data frame lasted 6.7 seconds, with every new frame available every 3.35 s. The DC component, the energy, the frequency-domain entropy, and the correlation coefficients were calculated for inclusion in the feature vector. In order to evaluate the entropy, the PDF of the STFT coefficients was estimated using an Epanechnikov kernel density estimator. Since five dual-axis accelerometers were considered in the experimental setup, each feature vector was composed of 30 components, which yielded the DC component, energy and entropy for the 10 data channels, plus 55 correlation coefficients (*d* = 85). Different selection algorithms were considered using the *k*-NN criterion. The maximum value for the criterion of selection was obtained by the SFFS method (Pudil algorithm), yielding an optimal subset of *d* = 17 feature components, amongst which 4 DC components and 13 correlation coefficients were found. The 17^th^-dimensional feature vectors were not submitted to any feature extraction step.

### 3.3. Single-frame classification algorithms

The single-frame classification algorithms included in Table 3 were trained and tested using data frames from the reduced dataset.

The figure of merit for classifier performance assessment was the aggregate classification accuracy; it was computed by constructing an aggregated confusion matrix that added the classification outcomes for all subjects. The algorithms were developed in MATLAB, using the PRTools [54] and the MatlabArsenal toolbox [55], which incorporates the Weka Machine Learning toolkit [56] and the LibSVM toolbox [57].

### 3.4. cHMM-based sequential classification algorithm

**Figure 5.**
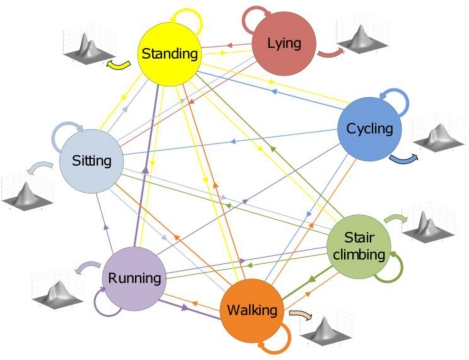
Sequential classification through an HMM-based classifier.

The cHMM-based sequential classification algorithm was a *Q*-state cHMM with continuous Gaussian emissions (*Q =* 7), Figure 5. The cHMM-based sequential classifier was developed using the HMM toolbox [58]. The similarity of this classifier to the single-frame classifier named GMM in Table 3 helped elucidating the merits of incorporating the statistical information provided by Markov modelling. An example of TPM that generates the OMM is reported in Table 4.

The classifier training was performed both by running the first-phase training only, and by combining it with the second-phase training, as discussed in Section 2.6. Additional testing was performed, where the TPM estimated in the first-phase was altered, before applying the cHMM-based classifier to incoming data during testing, both with and without the second-phase training.

Finally, additional testing was performed with the aim to specifically assess the classifier capability of protecting itself from spurious data. The threshold for spurious frame rejection was determined by performing a ROC study of sensibility and specificity of the classification process, averaged over all subjects. If not flagged as spurious, each feature vector presented to the classifier was assimilated by the Viterbi algorithm, used for estimating the most likely state sequence generated by the cHMM.

## 4. Results

The training set for single-frame classifiers is composed of *K* frames per class and per subject. According to the results of some preliminary testing, not shown here, *K* = 7 is a convenient choice for most single-frame classifier, in the face of the limitations imposed by the size of the reduced dataset. Testing is performed using the remaining *N*–*K* frames available for each subject.

The performance of the single-frame classifiers is reported in Table 5. The number of Gaussian components of the mixture is *M* = 1, either in the GMM or the cHMM-based classifiers. Preliminary testing of these algorithms up to *M* = 5, the results of which are not shown here, indicate only marginal improvements over the simpler choice *M* = 1 discussed in the following.

**Figure 6.**
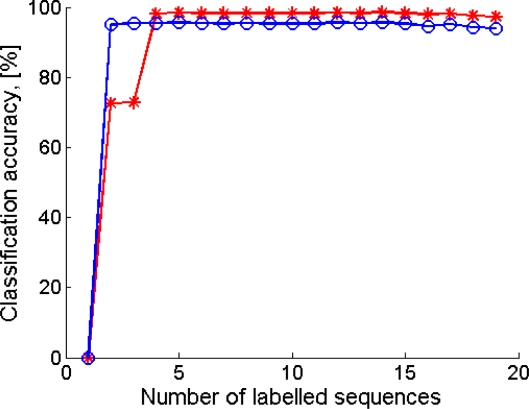
Classification accuracy *vs.* number of *P* of motor sentences in the training set. o: only first-phase training is applied; *: first-phase training is followed by second-phase training.

As for the cHMM-based sequential classifier, we settled *K* to the same value as for the single-frame classifiers (*K* = 7). The effect of the number *P* of motor sentences in the training set is then analysed and results are shown in Figure 6, either in the case that the second-phase training is performed or not, yielding *P* = 5 as a reasonable value for sizing the training set.

Results summarising the performance of the cHMM-based sequential classifier are given in Table 6. At the end of the first-phase of the learning process the testing is performed when the Baum-Welch algorithm is either applied or not.

**Figure 7.**
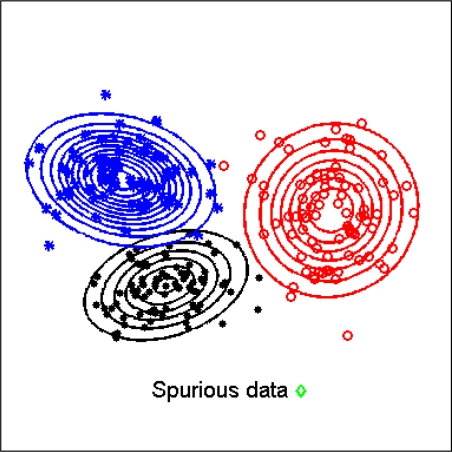
Feature vectors of three different classes are projected in a bi-dimensional subspace, to show how spurious data can be rejected based on the value of its likelihood.

Finally, we are interested in assessing the benefits of the rejection of spurious feature vectors, outlined in the previous Section (sensibility: 96.4%; specificity: 93.7%), Figure 7.

The performance improvement is remarkable yielding results similar to those achieved when spurious data frames are not inserted in the sequences to be classified, see Table 7.

## 5. Discussion and Conclusions

The classification accuracy achieved by analysing the acceleration reduced dataset for the purpose of classifying the seven primitives of Table 2 is remarkably high for all tested classifiers, either single-frame or sequential. Our results agree with the findings by [32], in spite of a slightly different approach to classifier construction. While they train each classifier by using examples from all tested subjects, we prefer to train separately one classifier for each subject, averaging the individual classification accuracies to yield the results shown in Tables 5, 6, and 7. We believe that a subject-specific training is important especially for the cHHM-based sequential classifier, because of the high mannerism exhibited by humans while performing a given physical activity.

It is remarkable that the features selected by the Pudil algorithm yield simply gross postural information (the DC components), and highlight the existence of stable patterns in the various acceleration time series due to coordinated motion of different body parts (the set of surviving correlation coefficients). Nonetheless, it is argued that energy and entropy time-domain features would be highly valuable, provided that we decide to investigate other activities, e.g., those from the set studied in [32] that have not been considered in this paper. Our decision to concentrate on the basic vocabulary of motor words shown in Table 2 is motivated by our ongoing work aimed at developing a wearable sensor system for pedestrian navigation [59,60].

An important contribution of this paper is the demonstration that Markov modelling can be an important weapon in our arsenal of computational methods for classification of human physical activity. In fact, it should be pointed out that the cHMM-based sequential classifier performs systematically better than its simple single-frame GMM counterpart (99.1% *vs.* 92.2%). Actually, the proposed sequential classifier wins over all its tested single-frame competitors (the best single-frame classifier, *i.e.*, the NM classifier ramps up to 98.5%). This highlights the relevance of exploiting the statistical knowledge about the human motion dynamics that is “trapped” within the Markov chain.

The supervised training is pursued in this paper with the idea to split the process of estimating the parameters of the cHMM-based sequential classifier into two distinct phases. This is a helpful recipe to effectively cope with the size limitations of the training set. *P* = 5 sequences lasting each just few minutes are enough to yield a suitable training set in the present application. In regard to this point, note that, when the second-level phase of training is not performed, the sequential classifier performance goes down to 95.6% from 99.1%, still superior to the single-frame GMM classifier performance, but inferior to few single-frame classifiers from Table 3.

A final point is related to the proposed method for managing spurious data. It is worthy noting that most published studies, including [32], handle the problem of the *fuzzy borders* by manual data cropping. In our approach, when a massive injection of spurious data is made in the generated sequences to be classified, the sequential classifier performance breaks down to 73.3% (without threshold-based detector). When the threshold-based detector is actually implemented the performance ramps up to 99.1%, which is slightly superior to the performance measured on “clean” data. It should be pointed out indeed that the criterion chosen to settle the threshold tends to remove spurious frames and even few noisy frames, the classification of which turns out to be wrong.

In conclusion, in this paper we have reviewed the various steps needed to implement a pattern recognition machine for automatic classification of human physical activity from on-body accelerometers. A major contribution of the paper lies in pursuing a Markov modelling approach to the design of one such machine. The results of extensive testing performed on an available dataset of acceleration time series shed light on the potential advantages of the proposed approach.

Future work will concern the integration of the proposed pattern recognition machine in the wearable sensor system we are currently developing in our lab for applications in the field of outdoor-indoor pedestrian navigation.

## Figures and Tables

**Table 1.** State of the art of human motor activity classification systems.

**Reference**	**Sensors**	**Features**	**Classifiers**	**Activity**	**Subjects**	**Accuracy [%]**
[38]	1 tri-axis accelerometer (3D acc)	Raw data Delta coefficients DC component	GMM	8	6	91.3
[39]	1 bi-axis accelerometer (2D acc)	Wavelet coefficients	k-NN	5	6	86.6
[40]	1 3D acc	Standard deviation Energy distribution DC component Correlation coefficients	Naive Bayesian k-NN SVM Binary decision	8	NA	46.3–99.3
[32]	5 2D acc	Standard deviation Energy distribution DC component Entropy Correlation coefficients	Naive Bayesian k-NN Binary decision	20	20	84
[34]	2 3D acc	Wavelet coefficients	ANN	4	6	83–90
[41]	1 2D acc	RMS velocity	ANN	6	10	95
[33]	1 2D acc Ambient sensors	Standard deviation FFT coefficients Derivative	ANN Markov chains	7	NA	42–96
[42]	1 3D acc	Wavelet coefficients Fractal dimension	Threshold-based	3	23	p < 0.01
[43]	1 3D acc	Wavelet coefficients	Threshold-based	3	20	98.8
[44]	1 2D acc 1 gyro	Wavelet coefficients	Threshold-based	5	44	> 90
[35]	1 3D acc	FFT	Threshold-based	9	12	95.1
[45]	1 2D acc 1 gyro 1 compass	Raw data Standard deviation Derivative	Threshold-based	5	8	92.9–95.9
[23]	2 uni-axis acc (1D acc)	Median Absolute deviation	Threshold-based	4	5	89.3
[19]	4 1D acc Heart and breath rate	FFT	Template matching	9	24	95.8
[30]	3 1D acc	DC component Standard deviation Signal morphology	Threshold-based Template matching	6	10	80–97.5
[46]	5 1D acc 1 2D acc	Angular signal Motility FFT	Binary decision	23	NA	81–93
[47]	1 3D acc	Magnitude area/vector Tilt angle FFT	Binary decision	10	6	90.8

**Table 2.** Activity primitives in the reduced dataset.

**Posture**	**Motion**
sitting	walking
lying	stair climbing
standing	running
	cycling

**Table 3.** Single-frame classifiers.

**Probabilistic approach**	**Geometric approach**	**Binary decision**
Naive Bayesian (NB)	Support vector machine (SVM)	Binary decision tree (C4.5)
Gaussian Mixture Model (GMM)	Nearest mean (NM)	
Logistic classifier	k-NN	
Parzen classifier	ANN (multilayer perceptron)	

**Table 4.** Example of TPM.

**Activity**	**lying**	**cycling**	**climbing**	**walking**	**running**	**sitting**	**standing**
**lying**	0.9500	0.0000	0.0000	0.0000	0.0000	0.0100	0.0400
**cycling**	0.0001	0.8999	0.0000	0.0400	0.0000	0.0100	0.0500
**climbing**	0.0001	0.0000	0.6199	0.2500	0.0100	0.0200	0.1000
**walking**	0.0001	0.0100	0.0300	0.7999	0.0200	0.0700	0.0700
**running**	0.0001	0.0100	0.0100	0.3500	0.3999	0.0100	0.2200
**sitting**	0.0200	0.0000	0.0100	0.0400	0.0000	0.8500	0.0900
**standing**	0.0100	0.0300	0.0100	0.1800	0.0300	0.1200	0.6200

**Table 5.** Single-frame classifier performance.

**Classifiers**	**Classification accuracy, [%]**
NB	97.4
GMM	92.2
Logistic	94.0
Parzen	92.7
SVM	97.8
NM	98.5
k-NN	98.3
ANN	96.1
C4.5	93.0

**Table 6.** Sequential classifiers classification accuracy.

**Training**	**Classification accuracy, [%]**
First-phase only	95.6
First and second-phase combined	98.4

**Table 7.** Performance in the presence of spurious data (one spurious frame every three data frames).

**Implementation**	**Classification accuracy, [%]**
Without rejection of spurious data	73.3
With rejection of spurious data	99.1
